# Chemo-immunotherapy for Older Patients with Chronic Lymphocytic Leukemia – Passé Yet?

**DOI:** 10.1097/HS9.0000000000000275

**Published:** 2019-07-05

**Authors:** Alexey V. Danilov, John M. Pagel, Jennifer R. Brown, Brian T. Hill

**Affiliations:** 1 Knight Cancer Institute, Oregon Health and Science University, Portland, OR; 2 Swedish Cancer Institute, Seattle, WA; 3 Department of Medical Oncology, Dana-Farber Cancer Institute, Boston, MA; 4 Department of Hematology and Medical Oncology, Cleveland Clinic, Cleveland, OH.

Chemo-immunotherapy regimens such as fludarabine, cyclophosphamide and rituximab (FCR), and bendamustine-rituximab (BR) have long been mainstays of therapy in chronic lymphocytic leukemia (CLL). However, in the past decade, recognition of the role of the microenvironment in the pathogenesis of CLL led to the development of targeted inhibitors of the B-cell receptor signaling-associated kinases.^1^ This resulted in a paradigm shift in treatment of CLL patients and significantly improved the natural history of the disease. The seminal paper published by Woyach et al from the Alliance for Clinical Trials in Oncology Cooperative Group further highlights the importance of targeted therapy in CLL.^2^ This randomized study of ibrutinib, a Bruton tyrosine kinase inhibitor, used as a single-agent or in combination with rituximab versus BR in patients ≥65 years old demonstrated a progression-free survival (PFS) benefit of a targeted agent over a commonly used standard chemo-immunotherapy in the front-line setting. While this study did not show an overall survival (OS) benefit, the results will impact CLL treatment practice on a global scale as ibrutinib continues to emerge as the preferred first-line therapy in older patients with CLL, independent of genetic risk features. This is not the first published evidence of ibrutinib superiority over chemotherapy. In the RESONATE-2 study, ibrutinib resulted in improved PFS and OS over chlorambucil in older patients,^3^ leading to its regulatory approval in the frontline setting in CLL.

Despite the advance of ibrutinib over chemo-immunotherapy in high-risk patients, several study details are worth highlighting. Importantly, not every patient may be ideally suited for frontline ibrutinib therapy, as the current standard of care has been to continue treatment indefinitely until progression or intolerance. In several studies, including Woyach et al, approximately 20% of patients discontinue the drug because of adverse events, even higher in real world analyses. Moreover, patients with comorbidities have been poorly represented on many clinical trials to date and are more likely to experience both dose reductions and interruptions, leading to shortened PFS and OS.^4^ Treatment with ibrutinib does not mitigate infectious risks, as up to 20% of patients on this study experienced grade ≥3 infectious complications, compared with 15% with BR. Aspergillus remains a problem.^5^ In addition, there were 4 instances of intracranial hemorrhage with ibrutinib, including 1 death.

Cardiovascular complications of ibrutinib have also been noteworthy. While less than 8% of patients experienced atrial fibrillation in this study, the incidence approaches 14% in a pooled analysis over a similar follow-up period.^6^ In addition, ventricular arrhythmias and sudden cardiac death have been described in ibrutinib-treated patients and this remains an unpredictable and potentially catastrophic event.^7,8^ Of note, in the current study, 12% to 13% of patients died of treatment-related side effects in the ibrutinib arms, compared with 9% on the control arm. Of 361 patients who received ibrutinib, 11 sustained ‘unwitnessed’ or unexplained deaths, including ‘sudden death’, ‘cardiac arrest’, ‘heart failure’ and ‘myocardial infarction’ over a follow-up period of 32 months. Only 2 such deaths occurred with 176 patients treated with BR. These unexpected and unpredictable cardiac toxicities should weighed when considering ibrutinib in frontline therapy of CLL. Meanwhile, BR remains an effective regimen, particularly in patients with favorable disease genetics, and has a lower rate of grade ≥3 non-hematologic adverse events (63% vs 74%) compared to the ibrutinib arms in the Woyach et al study. Lastly, the financial impact of incorporating novel agents into the front-line setting should not be ignored as more widespread use of these drugs dramatically raises the cost of managing CLL for both the society and individual.^9^ Given the above reservations, we suggest that the days of chemo-immunotherapy are not yet over.
